# Genetic and Molecular Basis of QTL of Diabetes in Mouse: Genes and Polymorphisms

**DOI:** 10.2174/138920208785133253

**Published:** 2008-08

**Authors:** Peng Gao, Yan Jiao, Qing Xiong, Cong-Yi Wang, Ivan Gerling, Weikuan Gu

**Affiliations:** 1 Departments of Orthopaedic Surgery, Campbell Clinic and Pathology, University of Tennessee Health Science Center, Memphis, Tennessee, USA; 2 Center for Biotechnology and Genomic Medicine, Medical College of Georgia, 1120 15th Street, CA4098, Augusta, GA, USA; 3 Department of Medicine, University of Tennessee Health Science Center, Memphis, Tennessee, USA

**Keywords:** Quantitative trait loci, type 1 diabetes, insulin-dependent diabetes mellitus (IDDM), candidate gene, polymorphism, mouse.

## Abstract

A systematic study has been conducted of all available reports in PubMed and OMIM (Online Mendelian Inheritance in Man) to examine the genetic and molecular basis of quantitative genetic loci (QTL) of diabetes with the main focus on genes and polymorphisms. The major question is, What can the QTL tell us? Specifically, we want to know whether those genome regions differ from other regions in terms of genes relevant to diabetes. Which genes are within those QTL regions, and, among them, which genes have already been linked to diabetes? whether more polymorphisms have been associated with diabetes in the QTL regions than in the non-QTL regions.

Our search revealed a total of 9038 genes from 26 type 1 diabetes QTL, which cover 667,096,006 bp of the mouse genomic sequence. On one hand, a large number of candidate genes are in each of these QTL; on the other hand, we found that some obvious candidate genes of QTL have not yet been investigated. Thus, the comprehensive search of candidate genes for known QTL may provide unexpected benefit for identifying QTL genes for diabetes.

## INTRODUCTION

Type 1 diabetes (T1D) is an autoimmune disease presenting with hypoinsulinemia, hyperglycemia, and ketoacidosis. It is also called insulin-dependent diabetes mellitus (IDDM) and juvenile-onset diabetes. Treating diabetes and its complications is a major drain on health care resources. Patients with T1D make up 5% to 10% of all cases of diabetes. In T1D patients, their own immune system damages the pancreatic ß cells in the islets of Langerhans, thereby abolishing endogenous insulin production. The exact causes of T1D are not well understood, although it is clear that genetic susceptibility plays a major role. There are more than 20 IDDM quantitative genetic loci (QTL) characterized so far.

Animal models have been widely used to study the genetics of human diseases. One of important uses of animal models is the mapping of quantitative trait loci. The term “QTL” is used for multiple genetic loci that control the same complex trait. A large number of QTL for type 1 diabetes have been identified in the NOD mouse model. However, the specific genes in those QTL that regulate the disease process have largely remained unknown. Because of the completion of mouse genome sequences, a relatively accurate list of all the genes within a chromosomal region defining a QTL can be obtained. In addition, recent rapid progress in gene expression profiles may provide clues to the relevance of many genes to type 1 diabetes. We decided to pursue potential candidate genes for QTL based on current genome resources and mapping information. 

We conducted a whole-genome search by using the Ensembl database (http://www.ensembl.org/index.html) and reports in PubMed. The candidacy of every gene in the region of each known QTL was evaluated based on published literature. The connection between a gene and type 1 diabetes was first established by searching for co-appearance of the name of a gene and specific key words in the literature. We first conducted a literature search in PubMed http://www.ncbi.nlm.nih.gov/entrez/query.fcgi?db=PubMed for potential candidate genes for each known QTL. For every potential candidate gene, up to 10 abstracts were read to confirm the relationship between this particular gene and T1D. Although some genes may play a role in autoimmunity, except for those IDDM loci without well characterized candidate genes, they were excluded if there was no supportive evidence for their role in T1D or ß cell function.

We examined 9038 genes from 26 QTL of type 1 diabetes. A total of 138 genes are considered to be candidate genes for those QTL. We produced a list of candidate genes for each of the known QTL, including several new and interesting genes that deserve further investigation.

## THE INFORMATION OF CURRENT QTL

### Information on QTL for Diabetes

A literature search was conducted with key words “diabetes” and “QTL” in PubMed for every publication through December 2007. Several QTL have been fine mapped with congenic breeding. Theoretically, the smallest genomic size should be the best for a candidate search of a QTL. We chose to provide candidate genes for every known locus, allowing investigators to make their own decision in choosing candidate genes for their studies and possibly to discover a closely linked gene with subtle interactive effects. Finally, we are aware that we may not have located every relevant publication or QTL by our search method.

### Information on Genes in QTL Regions

Genes within a QTL region were obtained from the Ensembl database. For fine-mapped and well-defined QTL, markers that flank the QTL were used for gene searching. For other QTL, a molecular marker at the peak region of the QTL was used as the middle point of the QTL. A genomic region of 10 megabase pairs (Mbp) around the peak marker was searched for candidate genes.

### Identification of Candidate Genes

For every known gene in a QTL, its potential connection with T1D was evaluated by searching information from the Online Mendelian Inheritance in Man (OMIM) (http://www.ncbi.nlm.nih.gov/entrez/query.fcgi?db=OMIM) and PubMed (http://www.ncbi.nlm.nih.gov/entrez/query.fcgi?db=PubMed). Search terms were a combination of the gene symbol with any of these seven key words: Type 1 diabetes, diabetes mellitus 1, beta cell, islets of Langerhans, insulin, autoimmune, or autoimmunity. These key words covered the disease, the target tissue of the pathogenic process, and the process itself. For any potential candidate, at least the abstract of one reference was read to determine the link between the gene and T1D. It is possible that the search terms could turn up other diabetes-related genes but not those with independent regulation of T1D. Nevertheless, because direct effects on T1D regulation may not yet be recognized for many genes, we chose to assemble a comprehensive list of potential candidate genes for consideration by interested scientists.

## CANDIDATE GENES FOR EVERY KNOWN QTL OF TYPE 1 DIABETES

### Candidate Genes for QTL of T1D on Chromosome 1

Chromosome 1 contains two QTL: *Idd26* and *Idd5*. We listed four QTL for chromosome 1 in Table **1**, however, *Idd5.1* and *Idd5.2* are two different versions of Idd5. Those two loci cover a total of 56,624,948 bp. Within those sequences there are 499 genes, from which we identified 13 as candidate genes using our search method.

The locus of *Idd26* contains 146 genes, among which zeta chain-associated protein kinase, *Zap70*, was the only candidate gene. It has been reported that in NOD mice, T cells are hyporesponsive to TCR-mediated stimulation because the PKC/Ras/MAPK pathway is blocked, which may be associated with targeting the Grb2/pp36-38/ZAP70 complex to the plasma membrane and cytoskeleton [1].

The other locus, *Idd5,* contains 353 genes from which cytotoxic T lymphocyte-associated 4, *ctla4*, has been reported in several studies [2-8] as the causal gene for this locus. In the mouse model of T1D, susceptibility was associated with a variation in *ctla4* gene splicing with reduced production of a spliced form lacking the CD80/CD86 ligand-binding domain [2]. A single nucleotide polymorphism (SNP) in *ctla4* exon 2 has been suggested as the genetic variation causing the biological effects of Idd5.1 [3]. *ctla4* RNA interference (RNAi) mice have a disease focused on primarily the pancreas, with rapid progression to diabetes, and the phenotype is major histocompatibility complex (MHC) dependent [4]. Optimal *Ctla* expression is controlled by a locus (*ctex*) telomere on chromosome 1 together with the *Idd3* (interleukin-2) gene upon CD3 activation of T cells [5].

While *Ctla4* appears to be the real causal gene, other genes such as *CD28, icos, slc11a1, irs1*, and *casp8* may also play roles in this important QTL, depending on different populations and genome backgrounds. Other studies reported several additional potential candidate genes. Dimorphism in intron 3 of CD28 was associated with T1D in early-onset patients [6]. Disruption of the CD28-B7 pathway and subsequent T_reg_ deletion restored autoimmunity, especially with increased incidence of diabetes in NOD-CD40L^-/-^ mice [7]*. *The *in vivo* costimulation by CD28 for inducing autoimmune disease strictly requires an intact C-terminal proline motif that promotes lymphocyte-specific protein-tyrosine kinase (LCK) binding to the CD28 cytosolic tail [8, 9]. But CD28 was excluded by fine mapping of the locus [3].

Another candidate in this region is *icos*. CD4^+^CD25^+^CD69^-^ T_reg_ operate directly in the autoimmune lesion and are dependent on inducible T cell costimulator (*icos*) to keep it in a nondestructive state [10]. Higher *icos* expression correlates with more IL-10 production by NOD-derived T cells, and this may be responsible for the less severe experimental autoimmune encephalitis (EAE) in NOD mice [11]. Again, no association with the microsatellite polymorphisms in the *icos* gene was found [6]. A polymorphism of *sclc11a1*(solute carrier family 11 member 1) influences the susceptibility to T1D in Japanese subjects [12] but not in European ancestry populations [44]. RNAi of *scl11a1* in NOD mice reduced the frequency of T1D, mimicking the protective *Idd5.2* region [13]. The G972R variant of IRS1 (insulin receptor substrate 1) was found to be associated with T1D, possibly because of its interaction with an unidentified locus on chromosome 8 [14], but this finding is unconfirmed [15]. NOD CD4^+^ T cells expressed higher levels of cell surface DAF/CD55 compared with NOD.Idd3/5 CD4^+^ T cells following activation with anti-CD3 and -CD28 [16]. Casp8 (caspase 8) is essential for ß cell apoptosis in type 1 and type 2 diabetes models and in regulating ß cell mass and insulin secretion under physiological conditions [17].

There are also several genes in this region related to pancreas development. Ligands of the ErbB-4 (v-erb-a erythroblastic leukemia viral oncogene homolog 4) receptors regulate the lineage determination of islet cells during pancreatic development along with EGF-R/erbB-1 signal [18]. Hh signaling, including Ihh (Indian hedgehog), is critical to both patterning in early pancreas development and in regulating insulin production in differentiated β cells [19]. HES6 (hairy and enhancer of split 6) can reverse nuclear reprogramming of insulin-producing cells following cell fusion [20].

### Candidate Genes for QTL of T1D on Chromosome 2

Chromosome 2 contains one QTL locus, *Idd13*. It covers 36,000,000 bp in a region between 105 Mbp and 141 Mbp. We found five candidates from a total of 437 genes (Table **1**). An important candidate is ß2-microglobulin (*b2m*). B2m is a serum protein found in association with the MHC class I heavy chain on the surface of nearly all nucleated cells. NOD-*b2m* null mice do not express cell surface MHC class I molecules or produce detectable levels of CD8^+^ T cells and are diabetes and insulitis resistant [21]. *Idd13* was found to work on nonhematopoietically derived cells controlling selection of diabetogenic T cells and/or their target pancreatic ß cells, and B2m-induced alterations in H2g7 class I conformation may partially explain these findings [22]. Another possible candidate is the synaptosomal-associated protein, 25-k; (*snap*-*25*). It has been reported that increased expression of *snap*-*25* is associated with nonesterified fatty acid (NEFA)-induced impairment of insulin secretion in mouse islets [23]. Decreased expression of *t*-*snare*, *syntaxin* *1*, and *snap-25* in pancreatic ß cells is involved in impaired insulin secretion from diabetic GK rat islets [24]. But most research on *snap*-*25* is related to type 2 diabetes (T2D). T1D and T2D likely share a final common pathway for ß cell dysfunction that includes secretion of IL-1ß and prostaglandins by immune effector cells, exacerbating existing ß cell dysfunction and causing further hyperglycemia [25]. However, whether *snap*-*25* should be considered a candidate is still in question, as the destruction of ß cells in T1D is very different from that in type 2 diabetes; one is caused by autoimmunity, while the other is caused by secondary destruction from hyperglycemia. The roles in T1D of other candidates such as *chgb* and *mrg* have not been confirmed.

### Candidate Genes for QTL of T1D on Chromosome 3

Three QTL on chromosome 3 cover 103,721,629 bp, which contain 1007 genes. Seventeen well-defined candidates were found from those QTL (Table **1**).

Both *Idd10* and *Idd17* are located on chromosome 3m and most of *Idd*17 is located within *Idd*10. Therefore, on the genome region of *Idd10* was analyzed. From *Idd10*, seven candidates were identified from a total of 618 genes. Toll-like receptor 2 (*Tlr2*) is one of the obvious candidates. During the stimulation of antigen-presenting cells and the development of autoimmune diabetes, sensing of ß cell death *via *TLR2 can be an initial event [26]. The *Tlr2* polymorphisms have been demonstrated to be associated with T1D and HLA independent [27]. A second candidate, *Cd101*, but not *Fcgr1 *(Fc receptor, IgG, high affinity I), has been reported to be responsible for the *Idd10* effect [28].

Four genes are secondary candidates because there is no direct association for these genes with T1D. Endosulfine-α (*Ensa*) is a weak candidate for its role in ß cell stimulation. Recombinant Ensa inhibits sulfonylurea binding to ß cell membranes, reduces cloned adenosine triphosphate postassium channel (K_ATP_) currents, and stimulates insulin secretion from ß cells [29]. Aristaless-like homeobox 3 (*Alx3*) is a candidate for its capability to regulate insulin gene expression in pancreatic ß cells [30]. Another autoimmunity-related gene is *Cd2* [31]. However, it is not clear whether it is associated with autoimmunity in addition to insulin secretion and resistance.

The second locus, *Idd*18, contains 321 genes. Four candidate genes were identified for this locus (Table **1**). *Ptpn22* (protein tyrosine phosphatase, nonreceptor type 22) may be the best described T1D gene. It is known that the 1858C/T allele of *Ptpn22* is the major risk variant for T1D, but an additional, infrequent coding variant at *Ptpn22* may also contribute [32, 33]. Besides *Ptpn22*, it has been reported that the presence of glutathione s-transferase mu-1 (*Gstm1*) may be a susceptibility factor in T1D for certain age groups [34]. Nuclear factor κB (*NF-κB*), subunit 1, which has been detected in numerous cell types, is another candidate gene. A case control study demonstrated significant association to T1D of certain *NF-κB* alleles in a United Kingdom population [35]. The frequency of the A7 allele of the *NF-κB1* gene is significantly increased in T1D adults, and an association of the AA genotype of *NF-κBIA* gene has been found for latent autoimmune diabetes in adults (LADA) [36]. However, no association for any allele of the *NF- κB1* microsatellite marker could be demonstrated in Danish T1D families [37].

Several other genes are not listed as candidate genes in Table **1** because of their negative evidence in T1D; these genes include pancreatic amylase (*Amy2*), vav3 oncogene (*Vav3*), and macrophage colony-stimulating factor 1 (*Csf1*). There was a distortion in the distributions of Amy2, but the result was not significant when corrected by performing multiple tests [38]. There is no evidence for *Vav3* polymorphisms associated with T1D [39]. Csf1 was also excluded from *Idd*18 by genetic interaction mapping [33].

The third locus, *Idd3*, contains 198 genes in its genome region. Six genes are considered to be candidates (Table **1**). Interleukin-2 and -21 are controversial candidate genes. Although NOD alleles at MHC (*Idd1*) and Il2 (*Idd3*) are not sufficient for T1D in the NOD mouse [40], the NOD allele of Il2 may functionally differ from the B6 allele in its ability to direct the transcription of activation-induced CTLA-4 [5]. NOD IIS-Idd3 congenic mice, which share the same alleles as the NOD mouse at both *Il2* and *Il21*, were indistinguishable from the NOD strain, indicating that both *Il2* and *Il21* can be candidates for *Idd3* [41]. Data suggest a contribution of IL21 and IL21R to a genetic susceptibility to T1D and possible involvement of IL-21 and its receptor system in the disease pathogenesis [42]. However, no association was found between polymorphisms of those genes and T1D in a Japaneses population [43].

Other candidates including *Foxo1*, *Gffr2*, and *Pld1* are not directly related to the T1D pathogenic process.

### Candidate Genes for QTL of T1D on Chromosome 4

Two independent QTL regions that contain 1519 genes within 79,815,215 bp: *Idd11* and *Idd9*. *Idd9.1, 9.2*, and *9.3* are different versions of *Idd9* (Table **1**). From those QTL, we found seven well defined candidates.

Three candidates were selected from a total of 722 genes at the *Idd11* locus: cell division cycle 42 (*Cdc42*), alkaline phosphatase 2 (*Akp2*), solute carrier family 2 (facilitated glucose transporter), and member 1 (*Slc2a1*). *Akp2*, a target gene of Runx2, was significantly down-regulated in insulin-deficient, hyperglycemic diabetic animals along with Runx2 itself and several other targets; however, insulin treatment of diabetic animals significantly restored their expression [44].

The C677T *Mthfr* (5,10-methylenetetrahydrofolate reductase) polymorphism does not have a significant role in the development of diabetic nephropathy in T1D [45, 46], although this gene was reported in type 2 diabetes development [47]. *Tnfr2* is also related to the regulation of autoimmune disease [48]. Three C1q genes, whose deficiency leads to autoimmunity, are located in *Idd*11 [49, 50, 51].

The role of *Lck* is controversial. Four SNP variants of *Lck* (lymphocyte protein tyrosine kinase) were frequently but not significantly associated with diabetes or LCK protein level. But of other SNPs studied, 11 were found only within the diabetic population and some were associated with low LCK protein levels. The low frequency of these polymorphisms did not permit any statistical significance, suggesting that the *Lck* gene probably does not contribute to the genetic susceptibility to T1D [52]. A major role for the common *Lck* polymorphisms in T1D is unlikely [53].

*Padi4* (peptidyl arginine deiminase, type IV) [54], *cdc42* [55], *Frap1* (FK506 binding protein 12-rapamycin associated protein 1), and *Slc9a1* (solute carrier family 9 member 1) [56] are not likely to be good candidates for T1D.

Candidate genes of *Idd*25 overlap with those of *Idd*9 and *Idd*11. *Idd*9 now has been narrowed to 335 genes; *Idd*25 may contain 402 genes. Four genes are considered to be candidates for this locus: tumor necrosis factor receptor superfamily, member 9 (*tnfrsf9*); histone deacetylase 1 (*Hdac1*); e2f transcription factor 2 (*E2f2*); and nuclear receptor subfamily 0, group b, member 2 (*Nr0b2*). *E2f2* is the best candidate in this region considering that mice deficient for both *E2F1* and *E2F2* develop nonautoimmune, insulin-dependent diabetes with high penetrance [57]. Another good candidate is *Tnfrsf9*. Functional analyses have demonstrated that purified T cells from NOD congenic mice with the C57BL/10 (B10) allele at *Idd9.3* produce more IL-2 and proliferate more vigorously in response to anti-CD3 plus immobilized 4-1BB/Tnfsf9 ligand than do T cells from NOD mice with the NOD allele at *Idd9.3.* In contrast, the response to anti-CD3 plus anti-CD28 costimulation was indistinguishable between the congenic strains, pinpointing the differences in NOD versus NOD.B10 *Idd9.3* T cell responses to the Tnfrsf9/4-1BB costimulatory pathway [58]. But once again, this gene was excluded for European ancestry populations [42].

### Candidate Genes for QTL of T1D on Chromosome 5

The genomic region of Idd15 includes 51 genes in a 10,000,000-bp region. No gene is currently reported to be directly related to T1D. However, it has been reported that T cells from semaphorin 3A (*Sema3A*)-deficient mice exhibited hyperproliferative responses to anti-CD3 stimulation and to allogeneic dendritic cells *in vitro* [59].

### Candidate Genes for QTL of T1D on Chromosome 6

Two QTL regions contain 817 genes in 74,962,203-bp genomic sequences. We found 24 candidate genes. 

The first region is *Idd19*, which overlaps with *Idd6*. CD4 and CD69 are among the obvious candidates. CD4^+^ cells play a key role in the pathogenesis of T1D. The percentages of naive T helper cells or suppressor/inducers CD4^+^ cells and CD4^+^CD45RA^+^ cells were increased in IDDM patients [60]. Decreased numbers, or function, of CD4^+^CD25^+^ T_reg_ cells have been linked to the development of T1D [61, 62]. TGF-α signaling in CD8^+^ T cells is critical for CD4^+^CD25^+^ T_reg_ cell suppression of islet-reactive CD8^+^ T cells in T1D [63]. CD69 is a negative modulator of autoimmune reactivity and inflammation through the synthesis of TGF- α, although the observation was noted on an arthritis model [64]. A lower level of CD25, CD71, CD69, and HLA-DR antigen expression was found in patients at all observation times and in pre-T1D subjects after administration of 1 µg/ml of PHA showed a significantly reduced expression of CD69 and CD71 [65]. In animal models of autoimmune diseases, self altered peptide ligands (APL) triggered up-regulation of CD69 and CD25 expression and efficiently down-regulated *in vitro* activation of a Th1 clone specific to the mitochondrial 38-kD islet antigen (Imogen) induced by either p55-70 epitope of Imogen or native ß cell auto-antigens [66]. BID (BH3 interacting domain death agonist) cleavage by granzyme B precedes mitochondrial disruption and apoptosis in pancreatic islets [67]. A large number of polymorphisms and amino acid changes were identified in both *Lrmp* (lymphoid-restricted membrane protein) and *Bcat1* (branched chain aminotransferase 1, cytosolic), indicating that they are candidates for *Idd6* [68]. The NOD allele at this locus mediates lower mRNA expression levels of the *Lrmp/Jaw1* [69]. But the rs2242400 polymorphism in *Bcat1* was associated with type 2 diabetes in more than one population [70]. In type 2 diabetes, the 825T allele was reported to be predispose for end-stage renal disease, whereas this effect has not yet been confirmed for patients with T1D [71]. *Arntl2* (aryl hydrocarbon receptor nuclear translocator-like 2) up-regulation correlated with the up-regulation of the ARNT-binding motif containing the *Pla2g4a* gene, which has recently been described as being protective for the progression of insulitis and autoimmune diabetes in the NOD mouse by regulating the TNF-α pathway [72]. Chemokine (C-X-C motif) ligand 12 (*SDF-1/Cxcl12*) can negatively regulate NOD/LtJ diabetogenic T cell adhesion, which may be important in regulating diabetogenic T cell recruitment into islets [73].

Some candidates do not have strong supportive data. Peptide IAPP (islet amyloid polypeptide) 9-17 is one of the HLA-A*0201-restricted T cell epitopes in T1D patients [74], but IAPP is more of a type 2 diabetes risk factor in the process of ß cell apoptosis [75]. There is an inverse relationship between GAPDH (glyceraldehyde-3-phosphate dehydrogenase) activity and Methylglyoxal production in both T2D and T1D [76]. Exocytosis of insulin depends on the regulation of SNARE complex assembly by WNK1 (WNK lysine deficient protein kinase 1)-Munc18c complexes [77]. *Lrp6* (low-density lipoprotein receptor-related protein 6) encodes a transmembrane protein that has 71% identity with, and is structurally similar to, a product of *Lrp5 *(low-density lipoprotein receptor-related protein 5), a proposed candidate gene for T1D [78].

Two genes have been ruled out. *Gnb3* (guanine nucleotide binding protein, ß 3) C825T polymorphism does not contribute substantially to an increased risk of developing end-stage renal disease (ESRD) [79]. *Lag3* (lymphocyte-activation gene 3) was also excluded [57]. *a2m* (α-2-macroglobulin) is unlikely to be a candidate considering that Ig allotypes (Gm, A2m, and Km determinants) were equally distributed in both diabetic patients and healthy controls [80].

The second QTL, *Idd*20, contains 276 genes. Candidates include Thyrotropin-releasing hormone (*Trh*), regenerating islet-derived 1 (*Reg1*), *Reg2*, *Pap/Reg3b* . The role of pituitary hormone is unclear. A case of acquired deficiency of pituitary GH, PRL, and TSH associated with T1D mellitus was reported [81]. But basal TSH concentrations were unchanged with or without metabolic control in patients [82]. HIP/PAP acts as a T cell auto-antigen in NOD mice [83] and becomes overexpressed in human diabetic islets because of the local inflammatory response. We did not find any association between abnormalities of either *reg1* *α* or *reg1* *β* gene with any type of diabetes we searched [84]. *Reg2* was upregulated in islets from NOD RIP-HuIFNβ mice at the onset of the autoimmune attack. IFNβ up-regulates *Reg1* and *Reg2* genes in NIT-1 cells. The overexpression of an IFNβ-induced auto-antigen (*Reg*) in the islets during inflammation might contribute to the premature onset of diabetes [85]. The autoimmune response against REG2 may convert a regenerative process into an islet-destructive process, thereby accelerating development of T1D [86].

### Candidate Genes for QTL of T1D on Chromosome 7

Two QTL are located on chromosome 7 and cover 1437 genes in 75,991,727 bp of the genomic region. Twelve well-defined candidates were identified from those two regions. 

From Idd7, three candidates were found from a total of 786 genes (Table **1**). *Galp* (galanin-like peptide) neurons are direct targets for insulin, and these cells play a role in the metabolic and behavioral sequelae of T1D [87]. Endogenous GALP provides trophic support to the neuroendocrine reproductive axis, including sexual behavior. T1D is associated with reduced expression of *Galp*, as well as an overall decline in reproductive function due to *Galp* [88]. *Ffar1* (free fatty acid receptor 1) was shown to be highly expressed in rodent pancreatic ß cells, and its down-regulation by RNAi caused impaired fatty acid stimulated insulin secretion [89]. Variants in the *Ffar1* gene are associated with ß cell function in T2D [90], but the role of this gene in T1D is unclear. Up-regulation of FLIP, caspase-8 inhibitor, enhanced NF-κB activity *via *NF- κB-inducing kinase and RELB can lead to increased PDX-1 and insulin production independent of changes in cell turnover [91].

Seven candidates were identified from 651 genes in the second locus, *Idd27*. Both the G866A polymorphism in uncoupling protein 2 (mitochondrial, proton carrier) (*Ucp2*) and the C55T polymorphism in *Ucp3* are associated with a reduced risk of diabetic neuropathy in T1D [92]. Inflammation is stronger in *Ucp2*-knockout mice and islets, leading to exacerbated diabetic conditions [93]. NOX4 (NADPH oxidase 4) is the major source of ROS in the kidneys during early stages of diabetes, and NOX4-derived ROS mediates renal hypertrophy and increased fibronectin expression [94]. Gene microarray analyses showed that α-GalCer treatment decreases IL-16 and increases IL-10 and *Mip1* *β* gene expression in the spleen. Anti-IL-16 antibody treatment protects NOD mice against insulitis and T1D [95].

Another IL-related protein, IL18BP (interleukin 18 binding protein), does not contribute to the overall genetic susceptibility to T1D because of low frequency of several polymorphism [96], but there is no direct evidence to exclude it. p21 (CDKN1A)-activated kinase 1 *Pak1* may mediate between *Cdc42* and *Rac1* in the pathway to transmit the glucose signal early in stimulus-secretion coupling to support the later stage of insulin release [97]. *Furin* and *St5* do not have strong supportive data.

### Candidate Genes for QTL of T1D on Chromosome 8

Chromosome 8 contains only one QTL, Idd22, which covers 25,000,000 bp, including 276 genes. Two well-defined candidates were found: oncogene jun-b (*Junb*) and interleukin-15 (*Il-15*). High IL-15 expression, detected in peritoneal macrophages of vitamin D-deficient mice, accelerates T1D development in NOD strain. The MAPK pathway may function through JunB in mediating some of the pleiotropic actions of secretagogues on pancreatic β cells.

Several other genes, including *Nod2, ccl17, ccl22, cx3cl1*, and CD97, are related to autoimmunity. The role of NOD2 in this IL-23-IL-1-IL-17 axis has been confirmed in NOD2-deficient DCs [98]. Although NOD2 is known in autoimmune Crohn's disease [99], there is no report on other autoimmune diseases. *Ccl17, ccl22, and cx3cl1*, three chemokine ligands, are also located in this region. Deletion of the *Daf*, ligand of CD97, exacerbates autoimmune disease development in MRL/lpr mice, a model for human systemic lupus erythematosus [100]. Ca^2+^-induced and cAMP-mediated potentiation of insulin secretion was unchanged in the absence of annexin A7 [101], thereby excluding the candidacy of this gene.

### Candidate Genes for QTL of T1D on Chromosome 9

Three candidates were identified from 346 genes in a 25,000,000-bp region of *Idd2* on chromosome 9 (Table **1**). In the biobreeding diabetes-resistant (BBDR) rat, KRV can induce innate immune activation and autoimmune diabetes through toll-like receptor 9 (TLR9)-signaling pathway [102]. Depletion of cellular PMR1 (ATPase, Ca^2+^-sequestering) with siRNAs inhibited Ca^2+ ^uptake into the endoplasmic reticulum and secretory vesicles by approximately 20%; subcellular fractionation of the cell lines revealed PMR1 immunoreactivity in both microsomal and dense-core secretory vesicle-enriched fractions [103]. Function of *Rhoa* in T1D has not been confirmed.

### Candidate Genes for QTL of T1D on Chromosome 11

*Idd*4 is located on chromosome 11 covering 36,000,000 bp and 732 genes. We examined 20 candidates. Plasma levels of glucagon-like peptide (GLP)-1 and gastric inhibitory peptide (GIP) did not differ between control and diabetic subjects [104]. IR-GIP seems not to be responsible for the changes in ß cell function after the onset of the disease [105]. In adult men with T1D, sex hormone-binding globulin (SHBG) was higher [106]; such was not the case in adolescents [107].

The function of a large group of chemokine genes in this region varies a lot in T1D. Adiponectin-mediated induction of IL-6, CCL2, and CXCL8 is disturbed in monocytes from T1D patients; therefore, elevated systemic adiponectin in T1D patients may be less protective [108]. The up-regulation of CCL3 and CCL4 *vs*. down-regulation of CCL2 suggests opposed functions of these chemokines in the disease process in T1D. No significant differences were seen for CCL5, CCL11, or CXCL10 [109]. CCR8/CCL1 interaction may play a role in T1D through macrophage recruitment and activation [110]. There was a marked decrease in transcripts of genes specific to β cells, followed by an increase in transcripts of chemokine genes (*cxcl1, cxcl5, and ccl7*) and of other genes typical of the myelo-monocytic lineages during disease progression [111]. Inflammatory responses that target islet ß cells are suppressed by CCL4 [112]. Pathogenicity was associated with T cell production of the macrophage-attracting chemokines CCL3 and CCL4 when T cells were primed with APCs expressing both B7-1 and ICAM-1 [113].

Besides those chemokines, a *Nos2* (nitric oxide synthase 2, inducible, macrophage) C/T single nucleotide polymorphism in exon 16 and resulting in Ser608Leu showed linkage to IDDM in HLA DR3/4-positive affected offspring [114]. But some populations do not have this association [115]. Insulitis was found in the islets from donors with serum positivity for islet cell antibodies (ICAs), glutamate decarboxylase aAbs (GADAs), insulinoma-associated protein 2 aAbs (IA-2As) and for the susceptible *HLA-DQ* genotype, and were consisted of CD3^+^/CD8^+^ T cells and CD68^+^ macrophages [116]. *Trpv1*^+^ (transient receptor potential cation channel, subfamily V, member 1) pancreatic sensory neurons control islet inflammation and insulin resistance. Eliminating these neurons in NOD mice prevents insulitis and diabetes [117]. Antimyeloperoxidase antibodies were detected in patients with T1D mellitus [118]. *Alox15, Alox12e, Psmb6, Pld2*, and *Cxcl16* are excellent candidate genes for the effects of the *Idd4* by fine mapping and quantitative real-time PCR [119]. 12/15-lipoxygenase (12/15-LO) and cyclooxygenase-2 (COX-2) pathways of arachidonate metabolism have been implicated in the pathogenesis of diabetic nephropathy [120].

Some other genes do not relate directly to T1D but to β cell function. Overexpression of the full-length Synip protein inhibited VAMP2 (vesicle-associated membrane protein 2) association with syntaxin 4 and decreased glucose-stimulated insulin secretion [121]. HNF-1β (HNF1 homeobox B) mutations may be associated with nondiabetic renal dysfunction and diabetes in Chinese and Italian patients [122, 123], but this gene is considered to be involved in T2D.

### Candidate Genes for QTL of T1D on Chromosome 13

*Idd14* covers 36,000,000 bp and 385 genes on chromosome 13, among which six candidates are mapped: *prl*, CD83, Bmp6, *Irf4*, *Syk*, and *cdkal1*. Prolactin PRL enhanced a Th2 response, which may reflect the preventive effect of PRL against development of multiple low-dose STZ diabetes in mice [124, 125]. The comparison of mature DCs between patients and controls indicated a difference in CD83 expression. The excess numbers of bone morphogenetic protein 6 (BMP6)-deficient myofibroblast progenitor cells may favor adverse tissue remodelling in patients [126]. *Irf4* (interferon regulatory factor 4) and *Tra1* with increased expression in B9-23 insulin peptide-treated NOD mice help promote the Th2 response [127]. Heightened IFN-α/β responses in NOD DC were not due to increased SYK kinase activity, excluding the candidacy of this gene. *Cdkal1* is more likely to be a T2D candidate.

### Candidate Genes for QTL of T1D on Chromosome 14

Two loci, *Idd*12 and *Idd*8, cover a genomic region of 44,717,940 bp and 388 genes on chromosome 14. Five candidates are listed in Table **1**. *Glud1* may be responsible for the effect of *Idd*12. Autosomal dominant mutations in the gene encoding glutamate dehydrogenase (GLUD1) lead to inappropriate insulin secretion by increasing the ATP/ADP ratio in the ß cells [128, 129]. Although BMPR1A is considered to function in glucose-stimulated insulin secretion, it is most likely to be a T2D-related gene.

So far, there is no report of *Idd*8 candidate genes. But IL-3 receptor is located within this region. *Pxk* [130] and *Dnase1l3* [131] are related to systemic lupus erythematosus but not to T1D.

### Candidate Genes for QTL of T1D on Chromosome 17

The search on chromosome 17 covers 37,262,334 bp and 843 genes. We found 22 candidates in three loci: *Idd*1, *Idd*16, and *Idd*23. 

*Idd*1 and *Idd*16 overlap each other. Candidate genes in these two regions were the same within our search. Pancreatic-derived factor (PANDER)-induced down-regulation of CDKN1A (cyclin-dependent kinase inhibitor 1A (P21)) expression coupled with induced CASP3-activation may serve a central role in islet cell death [132]. The ratio of cyclinD2/Cdkn1a, genes that respectively promote or inhibit cell cycle progression, was decreased in BioBreeding diabetes-prone (BBdp) islets [133]. Another nonclassical MHC class IB molecule, Qa-1, encoded by *H2-T23* is capable of presenting antigens to αβ and γδ T cells, and lymphocytes restricted to Qa-1 may contribute to immunoregulatory functions [134]. The third candidate is *Bat2* (HLA-B associated transcript 2). Microsatellite polymorphism in *Bat2* is associated with the age-at-onset of IDDM and possibly with the inflammatory process of pancreatic ß cell destruction during the development of IDDM. However, this association is not independent of TNFα polymorphisms [135]. HNF-1α haploinsufficiency in MODY3 subjects leads to decreased serum protein concentrations of APOM (apolipoprotein M), a proposed candidate of T1D [136].

*Idd*23 has three well-characterized candidates. *Pdcd2* (programmed cell death 2) is a likely susceptibility gene for T1D within this locus; two hypermorphs have been associated with a higher risk risk of T1D development [137]. Genes encoding the enzymes (superoxide dismutase 2, mitochondrial) Mn-SOD and extracellular superoxide dismutase (EC-SOD) were found to be associated with the pathogenesis of diabetic polyneuropathy (DPN) [138]. Maternal alleles at an *Igf2r* (insulin-like growth factor 2 receptor) polymorphism are associated with T1D [139]. Other genes including *tbp*, *psmb1, dll1, phf10, slc22a2*, and *slc22a3* do not have any supportive data.

### Candidate Genes for QTL of T1D on Chromosome 18

*Idd*21 covers 36,000,000 bp on chromosome 18, among which 301 genes are located. CD14 is the only gene reported to trigger autoimmune T1D in the NOD mouse [140]. Other genes including *apc* and *spink5* do not have supporting evidence.

## CONCLUSION

We conducted a comprehensive candidate search of reported T1D QTL identified from mouse models based on published genomic information, literature, and association of polymorphism with T1D. Our search revealed that a huge number of candidate genes exist within QTL regions. For most of them, careful studies regarding their relation to T1D QTL need to be done.

Our data also support the importance of maintaining the genome databases at a high level of currency. Most of the candidates selected based on the genetic map have not been identified as candidates in early studies. The simple explanation is that candidates in original publications were based on early, incomplete genetic information that does not accurately reflect the optimum location of genes.

For well-defined QTL, two markers flanking the QTL were used for gene searching. For QTL with more ambiguous boundaries, we searched the genome region that included 10 Mbp on each side of the molecular marker in the peak region of a QTL. We used this method, assuming that 10 Mbp on each side of peak marker provided enough genome sequences to encompass the genes underlying the QTL regions. We realize that this may not be true for every QTL. For the major QTL with large effects, there is a possibility that we did not cover all possible candidate genes. 

Our data also reveal the complexity in searching for candidate genes for T1D, as T1D is influenced by numerous genetic and environmental factors. For example, the destruction of β cells in T1D is very different from that of type 2 diabetes; one is by autoimmunity, while the other is by secondary destruction by hyperglycemia. Thus, a gene plays role in secondary destruction by hyperglycemia may not be relevant to T1D. Another question is that a candidate gene detected in mice may not have the same function or the effect may differ in humans. Furthermore, the richness in genetic alleles likely to characterize human gene regulation is more limited among inbred strains of mice due to common ancestry. Clearly, this assists initial mapping studies and eventual gene identification, while missing some molecular differences related to alleles. Nevertheless, we believe a combination of data from mouse models and human studies (population association, families, etc.) is a practical way to focus on the candidate genes revealed in this study. 

Fortunatley, we are in an era of rapid genomic research. Genome resources and technologies progress at an ever-increasing pace. In particular, the increasing comparative genomic information can complement mouse and human data by providing new insights to the diversity of a given gene's functions. We feel that a comprehensive search of candidate genes using updated genome information for known T1D QTL may provide unexpected benefits for our knowledge of the molecular bases and pathways of T1D.

## Figures and Tables

**Table 1:** Candidate Genes in Insulin Dependent Diabetes Loci in Mouse Genome

QTL	Total bps	Total Genes	Chr.	Searched Region (bp) or Markers		Total Genes	Candidate Genes*
Idd26	56624948	499	1	19797184	40207104	146	***Zap70***
Idd5			1	57726214	93726214	353	***Ctla4, Cd28**, Icos, ****Slc11a1****, **Irs1**, Ptprn* [141], *Acadl* [142], *Erbb4 *[18], *Ihh*, *Hes6*, *Klf7* [143] ***Casp8***
Idd5.1			1	57653560	93653560	352	
Idd5.2			1	57511186	93511186	352	
Idd13	36000000	437	2	105627827	141627827	437	***B2m***, ***Snap25**, Chgb* [144] , **Il1b*, Mrg1* [145]
Idd10	103721629	1007	3	73515038	109515038	618	***Tlr2**, Cd2* [31], *Ensa* [29], *Txnip, Slc16a1* [146] , *Alx3*
Idd17			3	67620523	103620523	535	
Idd18			3	99342152	135342152	321	***Ptpn22***, *Nfkb1, **Gstm1**, Pitx2* [147]
Idd3			3	20075190	56075190	198	***Il2***, ***Il21**, Foxo1* [148] , ***Slc2a2**, Fgf2* [149] , *Pld1*
Idd11	79815215	1519	4	113524850	149524850	722	*Cdc42, **Akp2**, Slc2a1*
Idd25			4	10Mbp from peak marker D4Mit71		402	
Idd9.1			4	110485381	146485381	668	*tnfrsf9* [44, 61, 150], *Hdac1* [151] , ***E2f2***, *Nr0b2* [152]
Idd9.2			4	124346244	154300596	575	
Idd9.3			4	130783882	154300596	446	
Idd9			4	135322552	154300596	335	
Idd15	10000000	51	5	3803678	13803678	51	*Sema3A*
Idd6	74962203	817	6	129942622	151104725	211	*lapp, Lrmp, Arntl2, Bcat1, Lrp6*
Idd19			6	111876879	147876879	519	***Cd4***, *Vwf, **Cd69**, Iapp, **Bid**, Gapdh, **Lrmp**, Gnb3, **Arntl2**, **Bcat1**, Lrp6, Cxcl12, Foxm1* [153] , *Pparg* [154] , *Wnk1*
Idd20							
			6	76142522	112142522	276	*Trh, **Pap**, **Reg1**, **Reg2***
Idd7	75991727	1437	7	1	35564320	786	***Apoe**, **Galp**, Ffar1, Akt2* [155] , *Relb*
Idd27			7	79249899	119677306	651	***Ucp2**, **Nox4, Ucp3, Il16**, Furin* [156] , *St5* [157] , *Pak1*
Idd22	25000000	276	8	74910379	99910379	276	*Junb* [158] , *Il15* [159]
Idd2	25000000	346	9	87254721	112254721	346	***Tlr9***, *Rhoa* [160] , *Atp2c1* [103]
Idd4	36000000	732	11	60764632	96764632	732	*Shbg, **Ccl2, Nos2, Ccl4, Ccl3**, Cd68, **Trpv1**, Ngfr, Mpo, **Ccl1**, **Ccl7**, Pld2, psmb6, **Cxcl16, Alox15**, **Alox12e**, Vamp2, Spop*, *Dusp14* [161] , *Hnf1b* [122, 162, 163]
Idd14	36000000	385	13	22900536	58900536	385	***Prl***, *Syk* [164] , *Cd83* [165] , *Bmp6, **Irf4**, Cdkal1* [166]
Idd12	44717940	388	14	8717940	44717940	321	*Glud1, Bmpr1a* [167]
Idd8			14	1	21444886	148	*Il3, Pxk, Dnase1l3*
Idd1	37262334	843	17	16741020	52741020	831	***Tnf***, *C4a* [168] , *C4b* [179], *Tap2* [169, 170] , *Tbp* [137, 171] , *Runx2* [172] , *Lta* [173, 174] , *Vegfa* [175] , *Nfkbil1* [176] , *Ager* [177], *Notch3* [178] , *Pim1* [179] , *Itpr3* [180] , *Cdkn1a*, *Srf* [181] , *H2-T23, **Bat2**, Apom*
Idd16			17	15478676	51478676	839	
Idd23			17	D17Mit113	Clcn7	254	***Pdcd2, Sod2, Igf2r***
Idd21	36000000	301	18	25051027	61051027	301	***Cd14***

* Bolded names are well-documented candidate genes.

## ABBREVIATIONS

APL: = Altered peptide ligands
DC: = Dedritic cell
DPN: = Diabetic polyneuropathy
EAE: = Experimental autoimmune encephalitis
ESRD: = End-stage renal disease
HLA: = Human leukocyte antigen
*Idd*M: = Insulin-dependent diabetes mellitus
K_ATP_: = Adenosine triphosphate postassium channel
LADA: = Latent autoimmune diabetes in adults
LCK: = Lymphocyte-specific protein-tyrosine kinase
MAPK: = Mitogen-activated protein kinase
Mbp: = Megabase pair
MHC: = Major histocompatibility complex
NEFA: = Nonesterified fatty acid
NF-κB: = Nuclear factor κB
OMIM: = Online Mendelian Inheritance in Man
PHA: = Phytohemagglutinin
PKC: = Protein kinase C
QTL: = Quantitative genetic loci
Ras: = Rat sarcoma virus oncogene 1
RNAi: = RNA interference
ROS: = Reactive oxygen species
SNP: = Single nucleotide polymorphism
TCR: = T-cell receptor
T1D: = Type 1 diabetes
T2D: = Type 2 diabetes
VWF: = Von Willebrand factor
